# Incidence and patterns of pediatric fractures at selected hospitals in Addis Ababa, Ethiopia, 2024: a multi-center cross-sectional study

**DOI:** 10.3389/fped.2025.1501900

**Published:** 2025-07-24

**Authors:** Sefa Muzemil, Amha Mekasha, Fetiha Muzemil, Muhiddin Tadesse Barega, Shamill Eanga

**Affiliations:** ^1^ Department of Pediatrics and Child Health, School of Medicine, St. Peter’s Specialized Hospital, Addis Ababa, Ethiopia; ^2^ Department of Pediatrics and Child Health, School of Medicine, Addis Ababa University, Addis Ababa, Ethiopia; ^3^ Department of Midwifery, College of Medicine and Health Sciences, Wolkite University, Wolkite, Ethiopia; ^4^ Department of Anesthesia, College of Medicine and Health Sciences, Wolkite University, Wolkite, Ethiopia

**Keywords:** fractures, incidence, pattern, pediatrics, hospitals, Ethiopia

## Abstract

**Introduction:**

Nearly 2,000 children under the age of 14 die every day due to injuries, with fractures accounting for 10%–25% of these cases. The burden of pediatric fractures is significant, but neglected and many can be prevented through simple and cost-effective community-level interventions. However, no studies have been conducted in our country regarding the incidence and patterns of pediatric fractures. Without comprehensive data, it is challenging to set priorities, formulate policies, and implement preventive measures. Therefore, this study aims to determine the incidence and patterns of pediatric fractures at selected hospitals in Addis Ababa in 2024.

**Materials and methods:**

A multi-center cross-sectional study was conducted from October 10, 2023, to February 15, 2024, in the emergency unit and pediatrics ward of three public hospitals in Addis Ababa, Ethiopia: Tikur Anbesa Specialized Hospital (TASH), Yekatit 12 Hospital, and Alert Hospital. Data was collected by bachelor's degree nurses using an interviewer-administered structured questionnaire. Data was refined, coded, and entered into Epi Info version 7 computer software, then exported into SPSS version 21 for data cleaning and analysis.

**Results:**

From a total of 648 children who encountered trauma, 182 cases were fractures, resulting in an incidence of 28% (95% CI: 26.5–29.7). The major patterns of injury were due to falls (79%), road traffic accidents (RTAs) (15.4%), and assaults (5.6%). The upper limb was the most common site of injury (73%). Fifty-four percent of the children had injuries to two structures.

**Conclusions:**

The overall incidence of pediatric fractures among those who encountered trauma was 28%. Male children exhibited a higher incidence of fractures than female children. The highest risk of fractures occurred in children aged 6–10 years. Upper limb fractures were observed more frequently than lower limb fractures.

## Introduction

Child injury is one of the most immediate public health threats, resulting in the deaths of nearly 2,000 children under the age of 14 every day around the world (1). Fractures are extremely common in the pediatric age group. Of all these injuries, fractures account for 10%–25% (2–4). The annual incidence rates of fractures have dramatically increased in children under 16 years, rising from 3.6 per 1,000–50 per 1,000. There was a doubled risk of fracture in boys aged 13–15 compared with their female peers. Typically, the incidence peaks at ages 11–12 for girls and 13–14 for boys. The lifetime risk of sustaining a fracture during childhood is approximately 42%–64% for boys and 27%–40% for girls; however, these gender distributions may vary between bone sites (5, 6). In children, the most common sites of injury are the upper limb, elbow, and forearm (7).

Activities associated with fractures were primarily soccer and bicycling. However, when comparing the total number of injuries associated with each activity, we found a doubled risk of fractures during rollerblading/skating or snowboarding (60%) compared to playing soccer (38%) or bicycling (33%) (8). Scaphoid fractures, which are infrequent in children, accounted for 9% of all fractures resulting from rollerblading/skating (9, 10). Injuries occur in various locations during different activities, and their patterns change over time. For instance, sports participation is encouraged to increase physical activity among children in most developed countries (11, 12). In contrast, other daily activities, such as walking, tend to decrease (13, 14).

A substantial proportion of pediatric fractures are preventable. In high-income countries (HICs), there has been a general decline in injuries over the past 15 years. However, children from low- and middle-income countries (LMICs) are disproportionately affected, accounting for over 90% of unintentional injury-related deaths globally (15, 16). In LMICs, efforts have largely focused on addressing mortality from communicable illnesses, resulting in a widening gap in injury mortality (17). The burden of pediatric fractures is significant, but many can be prevented through simple and cost-effective community-level interventions aimed at children and their families (1). The Ethiopian health sector program prioritizes the prevention of injuries and violence. To address pediatric trauma, the Ministry of Health (MoH) has been implementing Emergency Triage Assessment and Treatment Plus (ETAT+) strategies (18).

Without understanding the causes of injuries, as well as the conditions and environments in which they arise at different developmental stages, it is impossible to identify risky behaviours that can be addressed with age-appropriate preventive strategies (19). To the best of our knowledge, few studies have been conducted in Ethiopia regarding the incidence and patterns of pediatric fractures. Without comprehensive data on pediatric fractures, it is challenging to set priorities, formulate policies, and implement preventive measures. Therefore, this study aimed to determine the incidence and patterns of pediatric fractures at selected hospitals in Addis Ababa.

## Materials and methods

A multi-center cross-sectional study was conducted from October 10, 2023, to February 15, 2024, in the emergency unit and pediatrics ward of three public hospitals in Addis Ababa, Ethiopia: Tikur Anbesa Specialized Hospital (TASH), Yekatit 12 Hospital, and Alert Hospital. All three hospitals serve as teaching facilities and referral centers at both the city administration and federal levels. TASH is the largest tertiary hospital in the country and functions as one of the orthopedic centers, managing approximately 1,000 pediatric fracture cases per year. Yekatit 12 Hospital (Y12H) attends to around 250 pediatric fracture cases in its emergency and pediatric surgical wards annually. Alert Hospital (AH) sees about 4,600 pediatric emergency admissions annually, with 8% of these being pediatric fractures.

All children under 15 years old who sustained trauma and were admitted to TASH, Y12H, and AH were considered as the source population. The study population included all children under 15 years old who visited and were admitted to the selected hospitals during the study period, provided their caregivers gave voluntary consent for participation, and met the inclusion criteria. Children who died on arrival, those without an attendant, and those who declined consent were excluded from the study.

### Sample size determination

Since no study has been conducted in Ethiopia on the incidence of pediatric fractures, the prevalence of pediatrics fracture among our population was assumed to be 50%. The sample size was determined using a single population proportion formula, with a 5% marginal error and a 95% confidence interval (CI). The initial sample size was calculated to be 384. However, since the number of children visiting the emergency and trauma wards of the selected hospitals is less than 10,000, a correction formula was applied, resulting in a recalculated sample size of 165. After accounting for a 10% non-response rate, the final sample size was adjusted to 182.

### Sampling techniques

From a total of 12 public hospitals in Addis Ababa providing pediatric fracture services, Tikur Anbesa Specialized Hospital, Alert Hospital, and Yekatit 12 Hospital were selected randomly. Proportional allocation was done, and 47, 75 and 60 pediatric patients were selected from TASH, Alert Hospital, and Yekatit 12 Hospital, respectively. Data was collected consecutively until the required sample size was achieved.

### Data collection tool and procedure

After giving one day of training, data was collected by six bachelor's degree nurses from the emergency unit at selected hospitals both on working and night time using an interviewer-administered structured questionnaire to get sociodemographic information, mechanism of injury, time of injury and place of injury. The site of injury and type of fracture patterns were described by clinical evaluation and radiologic investigations. The questionnaire was initially prepared in English. The English version was translated into Amharic and translated back into English to ensure internal consistency. The questionnaire was developed from similar studies conducted in different countries (18–20).

### Data quality control

To ensure data quality, questionnaires were pre-tested with 5% of the sample size at St. Paul Hospital, and necessary amendments were made based on the findings. The investigator and supervisor checked the completeness of the data, and timely corrections were done

### Data processing and analysis

Data was refined, coded, and entered into Epi Info version 7 computer software, then exported into SPSS version 21 for data cleaning and analysis. Descriptive statistics were used for socio-demographic and clinical data. The incidence and pattern of pediatric fracture variables were analyzed using the Chi-square test.

### Ethical approval and consent to participate

This study was carried out under the Declaration of Helsinki Ethical Principles for Medical Research involving human subjects protocol (21). Ethical approval was received from the Ethical Review Board of Addis Ababa University, College of Medicine and Health Science with protocol number SOM/23/2024 and informed oral consent were taken.

## Results

### Sociodemographic characteristics

From 648 total pediatrics traumas in selected hospitals, 182 were diagnosed as fractures. Three -fourths of them were male, and 37.4% of the participants were in the age group of 6–10 years, with a mean and SD of 7.75 ± 3.97 years. Half of the participants had a family size of 4–5 members. One-third of the children received primary care from their mothers, and 28.6% of the mothers were housewives. Around 41% and 48% of the mothers and fathers completed higher education, respectively. The majority of the participants were from urban areas, and half of the pediatric families had a monthly income of 5000–10000 ETB (Table 1).

**Table 1:** The sociodemographic characteristics of the children and their families among pediatric fractures in selected hospitals in Addis Ababa, from October 10, 2023, to February 15, 2024 (*n* = 182).

Variable	Frequency	Percent
Sex of the children		
Male	131	72%
Female	51	28%
Age of children (years)		
<2	14	7.7%
2–5	42	23.1%
6–10	68	37.4%
>10	58	31.9%
Family size		
≤3	66	36.3%
4–5	93	51.1%
>6	23	12.6%
Care giver of the child		
Mother and Father	56	30.8%
Mother only	60	33.0%
Father only	4	2.2%
Others	62	34%
Maternal occupation		
Government employee	50	27.5%
Farmer	7	3.8%
Merchant	36	19.8%
Daily laborer	26	14.3%
House wife	63	34.6%
Maternal education		
No formal education	17	9.3%
Primary	28	15.4%
Secondary	62	34.1%
College and above	75	41.2%
Paternal education		
No formal education	7	2.8%
Primary	31	17.0%
Secondary	57	31.3%
College and above	87	47.8%
Residence		
Urban	158	86.8%
Rural	24	13.2%
Family monthly income		
<2,000	8	4.4%
2,000–5,000	40	22.0%
5,000–10,000	93	51.1%
>10,000	41	22.5%

### The incidence of pediatrics fracture

The findings of the study showed that out of the total of 648 children who encountered trauma in the emergency and trauma centers of the selected hospitals during the study period, 182 cases were fractures, making the incidence of pediatric fractures about 28% (95% CI; 26.5–29.7).

### The characteristics and pattern of injury

The major pattern of injury was falls, accounting for 79% of cases, followed by road traffic accidents (RTA) at 15.4%. In terms of injury mode, bicycle riding was responsible for 60.8% of the cases, while pedestrians accounted for 25%. Regarding the location of falls, 41.4% occurred in playgrounds, and 34.3% took place at home. Additionally, more than half of the children sustained injuries to two structures. Seventy-eight percent of the children had a single fracture, while 22% experienced multiple fractures (Table 2).

**Table 2:** The pattern of injury and fracture characteristics among children in selected hospitals in Addis Ababa, Ethiopia, from October 10, 2023, to February 15, 2024 (*n* = 182).

Variable	Frequency	Percent
Pattern of injury		
RTA	28	15.4
Falls	140	77
Physical child abuse	7	3.8
Metal injury	2	1.1
Fighting	5	2.7
Mode of injury in RTA victims		
Motorcycle	2	7.1
Bicycle	17	60.8
Pedestrian	7	25
Car accident	2	7.1
Place of fall down accident		
Home	48	34.3
Playground	58	41.4
School	34	24.3
Presence of two structure injured		
Yes	99	54.3
No	83	45.6
The pair of two structure injured		
Head, neck and lower extremities	2	1.9
Head, neck and upper extremities	4	3.8
Upper and lower extremities	93	88.6
Presence of two types of injury		
Yes	112	61.5
No	70	38.5
List of the injured type		
Fracture and STI	97	86.6
Fracture and dislocation	15	13.4
Number of fractures		
Single	141	77.5
Multiple	41	22.5

### The characteristics of limb fracture patterns and fracture sites

**Figure 1:**
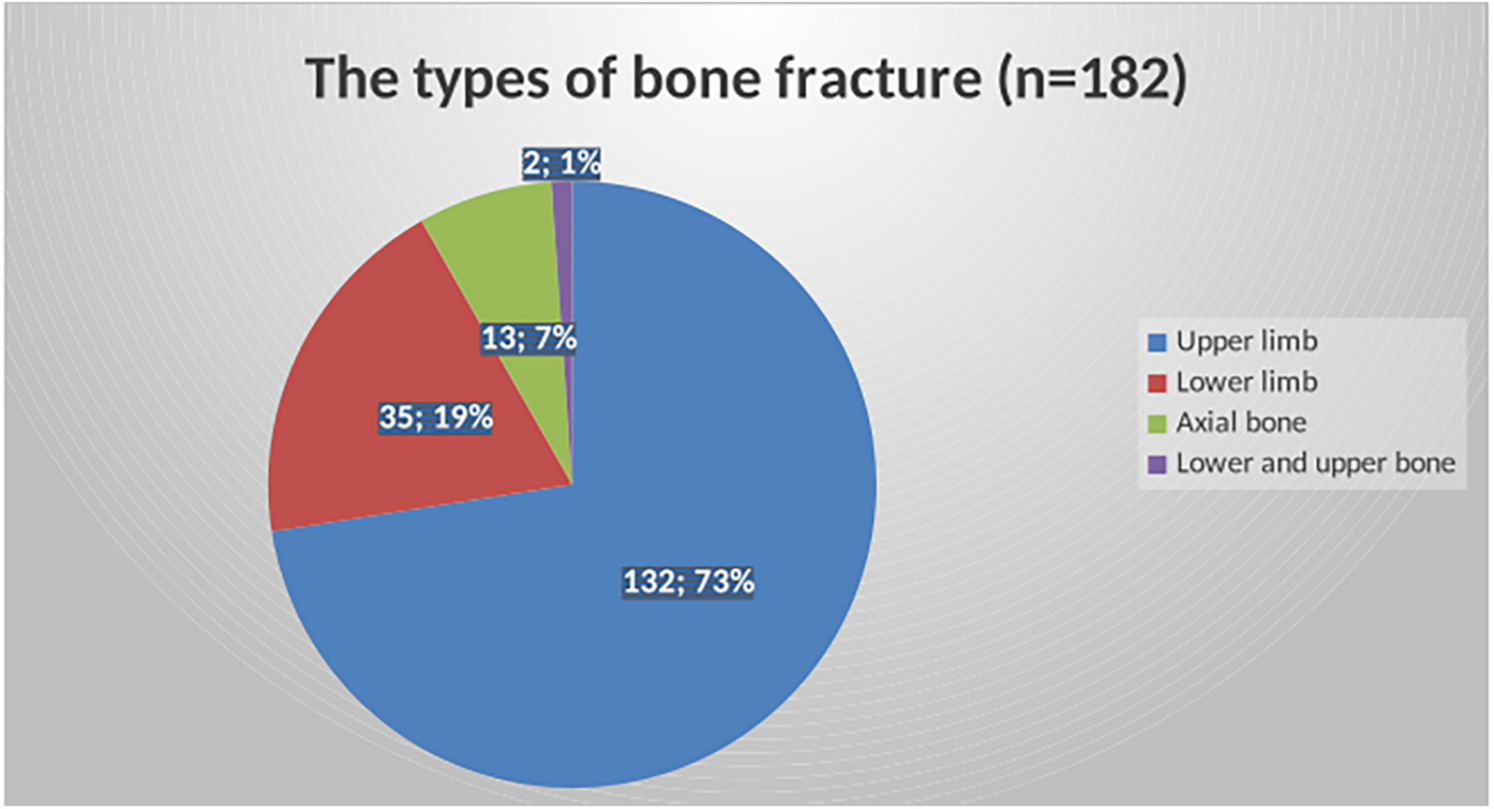
The patterns of fracture based on site among children in selected hospitals in Addis Ababa, Ethiopia, from October 10, 2023, to February 15, 2024 (*n* = 182).

From the total of 182 pediatric fractures, the upper limb accounts for seventy-three percent, the lower limb for 19%, and is followed by axial bone fractures (7%) (Figure 1). Thirty-nine percent of upper limb fractures were in the radius, followed by the ulna (26.7%), humerus (15%), and phalanges (7.4%). The majority of lower limb fractures were femoral fractures (55%), followed by tibial fractures (34%). Most of the fractures were of the closed (simple) type (Table 3).

**Table 3:** Characteristics of fracture sites among children in selected hospitals in Addis Ababa, Ethiopia, from October 10, 2023, to February 15, 2024 (*n* = 182).

Variable	Right side	Left side	Both side	Simple	Compound	*N* (%)
Lower limb						
Femur	11	10		18	3	21 (55.3%)
Tibia	5	8		11	2	13 (34.2%)
Fibula		4		4		4 (10.5%)
Upper limb						
Humerus	13	11		24		24 (15%)
Clavicle	12	1		13		15 (9.3%)
Radius	21	43		58	6	64 (39.7%)
Ulna	12	31		40	3	43 (26.7%)
Carpal	2			2		2 (1.2%)
Phalanges	8	3	1	9	3	12 (7.4%)
Meta carpal	1				1	1 (0.6%)
Axial bones	Types bone					
Skull	Linear			7		13 (7.1%)
	Compound			2		
	Temporal bone			5		
Facial	Nasal bone			7		8 (4.4%)
	Mandibular			1	1	
Sternum				1		1 (0.55%)
Ribs				1		1 (0.55%)

### The relation between patterns of injury with sex and age of the children

**Figure 2:**
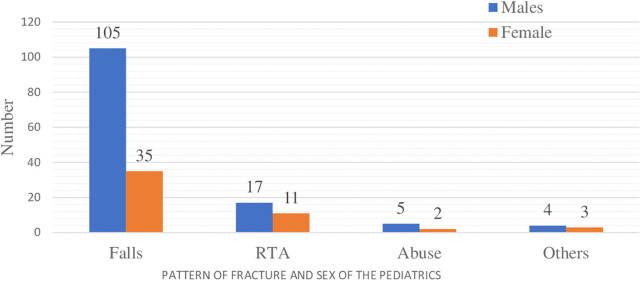
The patterns of fracture and sex of the children in selected hospitals in Addis Ababa, Ethiopia, from October 10, 2023, to February 15, 2024 (*n* = 182).

The findings of this study showed that males have a higher incidence of fractures (72%) compared to females (28%) due to falls, road traffic accidents (RTAs), and assaults, which account for 79%, 15.4%, and 5.6% of the cases, respectively (Figure 2).

The chi-square test was conducted to assess the association between sociodemographic characteristics and the incidence of fractures. The findings indicated that children in the 6–10 age group and those from urban residences had a higher incidence of fractures, which was statistically significant (*p*-value <0.05) (Table 4). Our results indicated that the RTA mode of injury (*p* = 0.014), place of fall (*p* < 0.001), two-structure injury (*p* = 0.034), and number of fractures (*p* = 0.012) were statistically significant in relation to the age of the children (Table 5).

**Table 4:** Association between sociodemographic characteristics and the incidence of fractures among pediatric patients in selected hospitals in Addis Ababa, Ethiopia, from October 10, 2023, to February 15, 2024 (*n* = 182).

Variable	Number of fractures		Chi-square value	*p*-value
	Single	Multiple		
Sex of the children				
Male	101	30	0.847	0.847
Female	40	11		
Age (years)				
<2	10	4	10.88	**0.012**
2–5	39	3		
6–10	54	14		
>10	38	20		
Care giver of the child				
Mother and Father	41	15	8.04	0.154
Mother only	49	11		
Father only	3	1		
Others	48	14		
Residence				
Urban	129	29	11.96	**0.001**
Rural	12	12		

Bold values are *p* < 0.05, indicates statistical significance.

**Table 5:** Association between pattern of injury and age of children among pediatrics in selected hospitals in Addis Ababa, Ethiopia, from October 10, 2023 to February 15, 2024 (*n* = 182).

Variable	Age in years				*p*-value
	<2	2–5	6–10	>10	
Pattern of injury					
RTA	1	7	12	8	0.204
Falls	13	25	62	40	
Abuse	0	2	5	4	
Metal injury	2	2	1	0	
Fighting	0	1	0	3	
Mode of injury in RTA victims					
Motorcycle	0	0	4	2	**0.014**
Bicycle	1	2	9	0	
Pedestrian	0	2	5	1	
Car accident	0	2	0	0	
Place of fall down accident					
Home	12	12	15	2	**<0.01**
Playground	1	8	25	22	
School	0	1	19	23	
Two structure injured					**0.034**
Yes	9	31	39	26	
No	5	11	29	32	
Number of fractures					
Single	10	39	54	38	0.012*
Multiple	4	3	14	20	

Bold values are *p* < 0.05, indicates statistical significance.

### Association between the fracture site and the sex of the children

The findings of this study showed that male children had a higher incidence of radial, ulnar, and skull fractures, which were statistically significant (*p* < 0.05) (Table 6).

**Table 6:** The association between fracture site and sex of the children among pediatrics in selected hospitals in Addis Ababa, Ethiopia, from October 10, 2023 to February 15, 2024 (*n* = 182).

Variable	Sex of the study participants		Chi-square vale	*p*-value
	Male	Female		
Femur	10	11	0.444	0.505
Tibia	12	1	0.68	0.411
Fibula	4			
Humerus	16	8	0.08	0.772
Clavicle	6	9	1.73	0.188
Radius	26	18	8.44	**0.004**
Ulna	32	11	5.72	**0.017**
Carpal	2			
Metacarpal	1			
Phalanges	12			
Skull	5	3	1.23	**0.012**
Ribs	1			
Mandibular		1		
Total site of fracture	134	62		

Bold values are *p* < 0.05, indicates statistical significance.

## Discussion

The current study found that, from 648 pediatric trauma children, the incidence of fractures was approximately 28% (95% CI: 26.5–29.7). Males have a higher incidence of fractures (72%) compared to females (28%), in relation to falls, RTA, and assaults, which account for 79%, 15.4%, and 5.6% of cases, respectively. This finding is consistent with studies conducted in Kenya (19), Italy (20), and the United States (22). However, our results differ from a study conducted at the Children's Hospital of Michigan, where females accounted for a greater proportion in the 10–12 year age group (23). This disparity in injury incidence may be attributed to age-related behavioral changes, such as males engaging in more physically risky activities.

The findings of the study indicate that children aged 6–10 years living in urban areas have a higher incidence of fractures. The mode of injury from RTA (*p* = 0.014), the place of fall (*p* < 0.001), two-structure injuries (*p* = 0.034), and the number of fractures (*p* = 0.012) were statistically significant in relation to the age of the children. These results are consistent with other studies conducted in South Wales (5) and Britain (6). The higher frequency of fractures may be attributed to increased participation in both organized and unstructured sports, as well as generally high levels of physical activity during this age.

Regarding the anatomic site, our results showed that the upper limb was more frequently involved, accounting for over 73% of fractures. This finding aligns with studies conducted at a Sub-Saharan tertiary care center (7) and in the United States (22). In contrast, other studies have reported a higher incidence of lower limb involvement (19, 20). The increased incidence of upper limb fractures in our study may be attributed to the fact that 79% of children reported falls as the main cause, with the upper limb being more commonly injured during such incidents. When individuals fall, they instinctively use their upper limbs to protect themselves, which increases the likelihood of fractures. The arm, in particular, may be frequently involved due to its role as a protective mechanism during a fall.

The other finding of our study indicated that the radius is more commonly involved in the upper limb, this is supported by several studies (6, 20). In contrast, another study conducted at TASH & Italy found that the humerus is more frequently involved (10, 18). These discrepancies may arise from variations in the distribution of fractures across different studies and populations.

In this study, falls accounted for 79% of fracture mechanisms, making them the most common, followed by road traffic accidents (RTAs). This finding aligns with other studies (3, 18, 19, 24). The high incidence of fall-related fractures may be attributed to the various activities in which children engage, such as climbing, running, jumping, and playing, all of which increase the risk of falls.

Our study showed that bicycle riding (60.8%) and pedestrian activity (25%) are the most common modes of fracture. Regarding the location of the falls, surrounding playgrounds and homes account for the majority of cases. This finding is consistent with other studies (9, 20, 23, 24) and reflects the amount of time that preschool children spend at home, which may be greater than their active time spent outside.

### Recommendations

#### For parents

Children should be actively supervised both at home and at playgrounds.

#### For health care providers

Health care providers should offer anticipatory guidance regarding home and playground injuries and advise parents on how to reduce these risks.

#### For policy makers

Policy makers should ensure that municipal policies on playground development and maintenance are based on established standards. Additionally, education on traffic rules and the construction of sidewalks, bridges, and tunnels to separate vehicles from pedestrians is recommended.

### Strength of the study

To the best of our knowledge, this study is the only prospective cross-sectional study conducted in multicenter settings in Ethiopia.

### Limitation of the study

Association was not done to identify risk factors of pediatrics fracture. Therefore, another strong study is needed to determine the incidence and risk factors of pediatrics fracture in the study setting to overcome the limitation of this study.

## Conclusions

The overall incidence of pediatric fractures among those who encountered trauma in selected hospitals during study period was 28%. Male children exhibited a higher incidence of fractures than female children. The highest risk of fractures occurred in children aged 6–10 years. Upper limb fractures were more frequently observed than lower limb fractures.

## Data Availability

The original contributions presented in the study are included in the article/Supplementary Material, further inquiries can be directed to the corresponding author.
